# Retinoic acid-induced 2 (RAI2) is a novel tumor suppressor, and promoter region methylation of RAI2 is a poor prognostic marker in colorectal cancer

**DOI:** 10.1186/s13148-018-0501-4

**Published:** 2018-05-23

**Authors:** Wenji Yan, Kongming Wu, James G. Herman, Xiuduan Xu, Yunsheng Yang, Guanghai Dai, Mingzhou Guo

**Affiliations:** 1 Department of Gastroenterology and Hepatology, Chinese PLA General Hospital, #28 Fuxing Road, Beijing, 100853 China; 2 Department of Oncology, Chinese PLA General Hospital, #28 Fuxing Road, Beijing, 100853 China; 3 Department of Oncology, Tongji Hospital of Tongji Medical College, Huazhong University of Science and Technology, Wuhan, 430030 China; 4 The Hillman Cancer Center, University of Pittsburgh Cancer Institute, 5117 Centre Ave, Pittsburgh, Pennsylvania 15213 USA

**Keywords:** RAI2, DNA methylation, Colorectal cancer, AKT signaling, 5-Aza-2′-deoxycytidine

## Abstract

**Background:**

Reduced expression of retinoic acid-induced 2 (RAI2) was found in breast cancer. The regulation and function of RAI2 in human colorectal cancer (CRC) remain unclear.

**Methods:**

Eight CRC cell lines and 237 cases of primary CRC were analyzed. Methylation-specific PCR (MSP), flow cytometry, xenograft mouse model, and shRNA technique were employed.

**Results:**

RAI2 was completely methylated in RKO, LOVO, and HCT116 cells; partially methylated in HT29 cells; and unmethylated in SW480, SW620, DLD1, and DKO cells. RAI2 was methylated in 53.6% (127/237) of primary colorectal cancer. Methylation of RAI2 was significantly associated with gender (*P* < 0.001), TNM stage (*P* < 0.001), and lymph node metastasis (*P* < 0.001). Analyzing by the Kaplan-Meier method, methylation of RAI2 was significantly associated with poor 5-year overall survival (OS) (*P* = 0.0035) and 5-year relapse-free survival (RFS) (*P* = 0.0062). According to Cox proportional hazards model analysis, RAI2 methylation was an independent poor prognostic marker for 5-year OS (*P* = 0.002) and poor 5-year RFS (*P* = 0.022). RAI2 suppressed cell proliferation, migration, and invasion and induced cell apoptosis in CRC. In addition, RAI2 inhibited AKT signaling in CRC cells and suppressed human CRC cell xenograft growth in mice.

**Conclusion:**

RAI2 is frequently methylated in human CRC, and the expression of RAI2 is regulated by promoter region methylation. Methylation of RAI2 is an independent poor prognostic marker of CRC. RAI2 suppresses CRC cell growth both in vitro and in vivo. RAI2 suppresses CRC by inhibiting AKT signaling.

## Background

Colorectal cancer (CRC) is the third most common cancer and the fourth most common cause of cancer-related death globally, accounting for roughly 1.2 million new cases and 600,000 deaths per year [1, 2]. The notion that aberrant epigenetic changes are involved in cancer development is widely accepted [3, 4]. In cancer, it has been demonstrated that gene expression is largely perturbed by disrupting the epigenetic machinery [5]. The recognition of an epigenetic component in tumorigenesis, or the existence of a cancer “epigenome,” has led to new opportunities for the understanding and detection, treatment, and prevention of cancer [6–8].

Retinoic acid (RA) plays an important role in development, adult hematopoiesis, and cell differentiation [9]. In fact, retinoid-based differentiation therapy of acute promyelocytic leukemia was one of the first successful examples of molecularly targeted treatment strategies [10]. The growth and differentiation of epithelial cells are strongly controlled by retinoid-activated genes. Retinoids are currently used as chemotherapies against cancers of epithelial origin. CRC is highly influenced by diet; therefore, it stands to reason that direct contact with retinoids from supplemented diets or exogenous retinoids administered as medication may have chemotherapeutic effects on CRC tumors [11]. Our previous study found that epigenetic disruption of retinol-binding protein 1 (CRBP1) and retinoic acid receptor β2 (RARβ2) is a common event in human cancers, including CRC [12]. Retinoic acid-induced 2 (RAI2) is located in human chromosome Xp22.13 [13, 14], a region in which microdeletion has been identified in Nance-Horan syndrome (NHS). This chromosomal region mainly includes four genes: REPS2, NHS, SCML1, and RAI2 [14, 15]. By screening eight familial cases and one sporadic case, no mutations or polymorphic sequence alterations were identified within RAI2 gene, and RAI2 has been excluded as disease-causing in NHS by this study [16]. The expression of RAI2 has been reported to be reduced in breast cancer [17]. By analyzing The Human Protein Atlas, we found that RAI2 was highly expressed in normal human colonic tissue samples, and its expression levels were reduced in colorectal cancer samples. RAI2 was rarely mutated in CRC according to The Cancer Genome Atlas (TCGA) database analysis. The regulation and function of RAI2 in CRC remain to be elucidated. Therefore, in this study, we focused on the epigenetic regulation and function of RAI2 in human CRC.

## Methods

### Primary human colorectal cancer samples and cell lines

A total of 237 cases of primary colorectal cancer were surgically resected, and 15 cases of normal colorectal mucosa were collected from non-cancerous patients by biopsy under endoscopy. Among the 237 cancer samples, 32 cases of paraffin blocks were available with matched adjacent tissue. All tissues were collected from the Chinese PLA General Hospital according to the approved guidelines of the Chinese PLA General Hospital’s Institutional Review Board. In addition, eight colorectal cancer cell lines (LOVO, SW480, HT29, RKO, HCT116, DLD1, SW620, and DKO) were included in this study. All colorectal cancer cell lines were previously established from primary colorectal cancer and maintained in 90% RPMI 1640 (Invitrogen, CA) supplemented with 10% fetal bovine serum. Cells were passaged 1:3 when total confluence (approximately 10^6^ cells) was reached in a 75-cm^2^ culture flask.

### 5-Aza-2′-deoxycytidine and MK2206 treatment

For methylation regulation analysis, colorectal cancer cell lines were split to low density (25% confluence) 12 h before treatment. Cells were treated with 5-aza-2′-deoxycytidine (DAC, Sigma, St. Louis, MO) at a concentration of 2 μM in the growth medium, which was exchanged every 24 h for a total 96-h treatment. At the end of the treatment course, RNA was extracted. To evaluate the effect of RAI2 on AKT signaling, DLD1 cell knockdown of RAI2 by shRNA was then treated by MK2206 (MedChemExpress, Monmouth Junction, USA), an AKT inhibitor. Cells were treated by MK2206 at 1 μM for 24 h for further study.

### RNA isolation and semi-quantitative RT-PCR

Total RNA was extracted using Trizol reagent; cDNA was synthesized according to the manufacturer’s instructions (Invitrogen, CA, USA). Glyceraldehyde-3-phosphate dehydrogenase (GAPDH) was used as control. The RAI2 PCR primer sequences were as follows: 5′-GCGATTGCGAGGCCAAGTG-3′ (forward) and 5′-GGGCCTTCTTTACCAGGTCAG-3′ (reverse). The GAPDH PCR primer sequences were as follows: 5′-GACCACAGTCCATGCCATCAC-3′ (forward) and 5′-GTCCACCACCCTGTTGCTGTA-3′ (reverse). Each experiment was repeated for three times.

### Bisulfite modification, methylation-specific PCR, and bisulfite sequencing

**Fig. 1:**
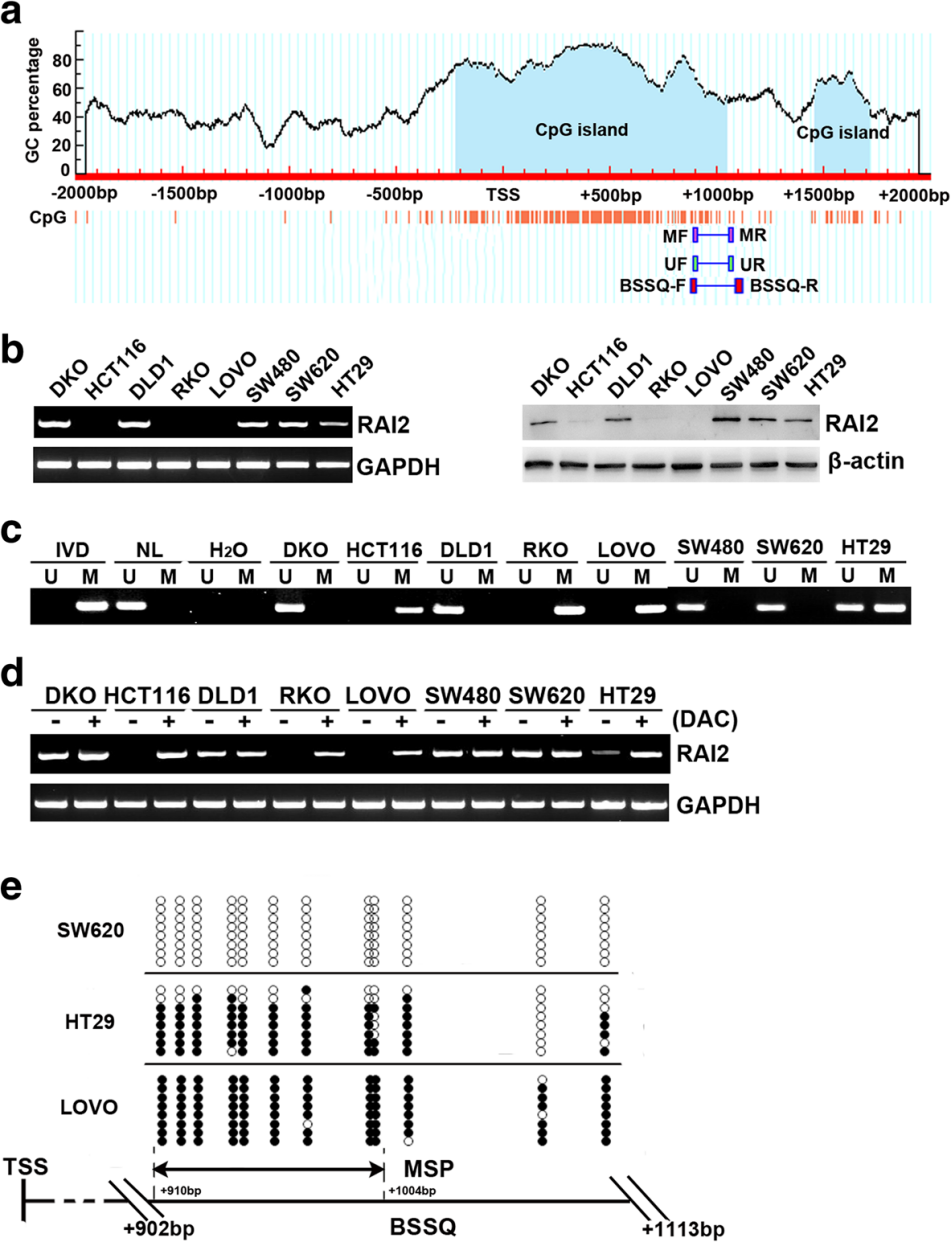
Expression of RAI2 is inactivated by DNA methylation in colorectal cancer cell lines. **a** Schematic diagram of CpG islands in the RAI2 promoter region. MF, MSP forward primer; MR, MSP reverse primer; UF, unmethylation forward primer; UR, unmethylation reverse primer; BSSQ-F, bisulfite sequencing forward primer; BSSQ-R, bisulfite sequencing reverse primer. **b** Expression of RAI2 was analyzed by semi-quantitative RT-PCR and Western blot in colorectal cancer cell lines (DKO, HCT116, RKO, HT29, SW480, SW620, LOVO, and DLD1). **c** Methylation status of RAI2 detected by MSP in colorectal cancer cell lines. IVD, in vitro-methylated DNA; NL, normal lymphocyte DNA; M, methylated alleles; U, unmethylated alleles. **d** Expression of RAI2 was analyzed by RT-PCR in colorectal cancer cell lines in the absence or presence of 2 μmol/l DAC (+) for 96 h. **e** Bisulfite sequencing of RAI2 was performed in SW620, HT29, and LOVO cell lines. The region of CpG islands studied by MSP is indicated by a double-headed arrow spanning 95 bp. Filled circles represent methylated CpG sites within the RAI2 CpG islands, and open circles denote unmethylated CpG sites. Bisulfite sequencing focused on a 212-bp (+ 902 to + 1113 bp) CpG islands downstream of the RAI2 transcription start site

Methylation-specific PCR (MSP) and bisulfite sequence (BSSQ) primers were designed in the locations of CpG islands in the promoter region of RAI2 gene according to MethyPrimer (Fig. 1a, http://www.urogene.org/cgi-bin/methprimer/methprimer.cgi). Genomic DNA from CRC cell lines and CRC tissue samples were prepared using the proteinase K method. Normal lymphocyte DNA was prepared from healthy donor blood lymphocytes by proteinase K method. Normal lymphocyte (NL) DNA was used as unmethylation control, and in vitro-methylated DNA (IVD) was used as methylation control. IVD was prepared using SssI methylase (New England Biolabs, Ipswich, USA) following the manufacturer’s instructions. MSP primers were designed according to genomic sequences inside the CpG islands in the RAI2 gene promoter region. MSP was performed as described previously [18]. MSP primers were designed according to genomic sequences around the transcription start site (TSS) in the CpG islands of the RAI2 gene promoter region and synthesized to detect unmethylated (U) and methylated (M) alleles. MSP was performed using 2720 Thermal Cycler (Life Technologies, Carlsbad, USA), and cycle conditions were as follow: 95 °C 5 min for 1 cycle; 95 °C 30 s, 60 °C 30 s, and 72 °C 30 s for 35 cycles; and 72 °C 5 min for 1 cycle. BSSQ products were amplified by primers flanking the targeted regions including MSP products. BSSQ was performed as previously described [19]. Cycle conditions were as follows: 95 °C for 5 min for 1 cycle; 95 °C for 30 s, 55 °C for 30 s, and 72 °C for 40 s for 35 cycles; and 72 °C for 5 min for 1 cycle. MSP primers were designed as follows:

5′-TTATGTTAGGTATCGAGTAACGTTTTC-3′ (M-forward), 5′-CGAAAAAAAACAACAACTCCCTCCG-3′ (M-reverse), 5′-TGTTATGTTAGGTATTGAGTAATGTTTTT-3′ (U-forward), and 5′-CACAAAAAAAAACAATAACTCCCTCCA-3′ (U-reverse).

BSSQ primers encompassed a 212-bp region downstream of the RAI2 transcription start site (+ 902 to + 1113 bp) and included the region analyzed by MSP. BSSQ primers were designed as follows: 5′-GGGTTTTTTGTTATGTTAGGTAT-3′ (forward) and 5′-ATAAAATACCATTTCCCCACC-3′ (reverse).

### Immunohistochemistry

IHC staining was performed on 4-μm thick serial sections derived from formaldehyde-fixed paraffin blocks. Rabbit polyclonal antibody against RAI2 (Abcam, ST. Louis, MO) was diluted at 1:30. IHC was performed and evaluated as described previously [20].

### Plasmid construction and transfection

The expression vectors for RAI2 were subcloned into Plenti6 lentivirus expression vector, and RAI2 expression lentiviral or empty vectors were packaged using ViraPower™ Lentiviral Packaging Mix (Invitrogen, CA, USA) to infect RKO and LOVO cell lines to establish stable expression cells. The infected cells were selected by blasticidin (Invitrogen, 461,120) at a concentration of 10 μg/ml. Lipofectamine 3000 (Invitrogen, L3000015) was used for plasmid transfection. For transient transfection, RAI2 CDS region was cloned into the pcDNA3.1 (+) plasmid (Era Biotech, Shanghai, China). All constructs were confirmed by sequencing.

### Knockdown of gene expression

Four shRNA molecules were designed to target all four transcripts of RAI2 and constructed into pGPU6/GFP/Neo vector (GenePharma, Shanghai, China). These shRNAs were then transfected into DLD1 cells according to the manufacturer’s instructions. The target sequences in the RAI2 gene were as follows: shRNA-1 (5′-GCTGTGCTCCAGAATTTGTTT-3′), shRNA-2 (5′-GCCACACGGTCATTAAGATGG-3′), shRNA-3 (5′-GGGAAGAGTCCATGGGAAATG-3′), and shRNA-4 (5′-GAAATACACATGCTCCCAATC-3′). The most effective construct, shRNA-2, was selected for the study.

### Colony formation assay

Cells were seeded at 1000 cells per well in 6-well culture plates in triplicate. The complete growth medium conditioned with blasticidin at 2 μg/ml was exchanged every 48 h. After 2 weeks, the cells were fixed with 75% ethanol for 30 min and stained with 0.2% crystal violet (Beyotime, Nanjing, China) for visualization and counting. Each experiment was repeated for three times.

### Cell viability detection

Cells were plated into 96-well plates at 1.5 × 10^3^ cells/well, and the cell viability was measured by the MTT assay (KeyGEN Biotech, Nanjing, China) at 0, 24, 48, and 72 h. Absorbance was measured on a microplate reader (Thermo Multiskan MK3, MA, USA) at a wavelength of 490 nm. Each experiment was repeated for three times.

### Flow cytometry

Staurosporine may induce apoptosis in cultured cells. To increase the sensitivity of apoptosis detection, RAI2 unexpressed and re-expressed RKO and LOVO cells were treated with staurosporine (STS) at 100 ng/ml for 24 h. DLD1 cells with or without RAI2 knocked down were treated with staurosporine (STS) at 120 ng/ml for 24 h. The cells were prepared using the Annexin V-FITC Apoptosis Detection Kit I (BD Biosciences, Franklin Lakes, NJ, USA) following the manufacturer’s instructions and then sorted by FACS Calibur (BD Biosciences, Franklin Lakes, NJ, USA). Each experiment was repeated for three times.

### Transwell migration assay and invasion assay

Cells were suspended in serum-free medium, seeded (1 × 10^5^) into the upper chamber of an 8-μm pore size transwell apparatus (Corning, NY, USA) and incubated for 20 h. Cells that migrated to the lower surface of the membrane were stained with crystal violet and counted in three independent fields. For invasion analysis, cells (1 × 10^5^) were placed into the upper chamber of a transwell apparatus coated with Matrigel (BD Biosciences, Franklin Lakes, NJ, USA) and incubated for 48 h. Cells that invaded into the lower membrane surface were stained with crystal violet and counted in three independent fields. Each experiment was repeated for three times.

### Western blot

Protein preparation and Western blot were performed as described previously [20]. The antibodies for Western blot analysis were as follows: rabbit anti-RAI2 (Cell Signaling Technology, Danvers, MA,USA), rabbit anti-MMP2 (Abcam, Cambridge, UK), rabbit anti-MMP7 (Abcam, Cambridge, UK), rabbit anti-MMP9 (Abcam, Cambridge, UK), rabbit anti-caspase 3 (Abcam, Cambridge, UK), rabbit anti-cleaved caspase 3 (Abcam, Cambridge, UK), rabbit anti-AKT (Bioworld Technology, Beijing, China), mouse anti-phospho-AKT^ser473^ (Cell Signaling Technology, Danvers, MA,USA), anti-E-cadherin (BD Biosciences, Franklin Lakes, NJ, USA), and anti-vimentin (Cell Signaling Technology, Danvers, MA,USA). Rabbit anti-actin (Cell Signaling Technology, Danvers, MA, USA) was used as a control. Each experiment was repeated for three times.

### Xenograft mice model

Stably transfected RKO cell line with pLenti6 vector or pLenti6-RAI2 vector (3 × 10^6^ cells in 0.15 ml phosphate-buffered saline) was injected subcutaneously into the dorsal right side of 4-week-old male Balb/c nude mice. Each group included five mice. Tumor volumes were measured every 3 days starting 7 days after implantation. Tumor volume was calculated according to the formula: *V* = *L* × *W*^2^/2, where *V* represents the volume (mm3), *L* represents the largest diameter (mm), and *W* represents the smallest diameter (mm). Mice were sacrificed on the 22nd day, and tumor weights were measured. All procedures were approved by the Animal Ethics Committee of the Chinese PLA General Hospital.

### Statistical analysis

The RNA sequencing (RNA-Seq) data for RAI2 gene expression in the dataset of CRC and normal tissues were downloaded from Genotype-Tissue Expression (GTEx) database (https://www.gtexportal.org/home/datasets) and the Cancer Genome Atlas (TCGA) (http://xena.ucsc.edu/, 08/16/2016), respectively. Statistical analysis was performed using SPSS 17.0 software (SPSS, Chicago, IL). Chi-square or Fisher’s exact tests were used to evaluate the relationship between methylation status and clinical pathological characteristics. The two-tailed independent samples *t* test was applied to determine the statistical significance of the differences between the two experimental groups. Survival rates were calculated by the Kaplan-Meier method, and the differences in survival curves were evaluated using the log-rank test. Cox proportional hazards models were fit to determine independent associations of RAI2 methylation with 5-year OS and 5-year relapse-free survival (RFS) outcomes. Two-sided tests were used to determine the significance, and *P* < 0.05 was considered to be statistically significant.

## Results

### RAI2 is silenced by promoter region hypermethylation in colorectal cancer cells

By analyzing The Human Protein Atlas, RAI2 was found to be highly expressed in normal human colonic tissue, and its expression was reduced in colorectal cancer samples (http://www.proteinatlas.org/ENSG00000131831). According to The Cancer Genome Atlas (TCGA) database analysis, in 224 cases of CRC samples, RAI2 was only mutated in three cases (https://cancergenome.nih.gov). To better understand the regulation of RAI2 expression in colorectal cancer, the levels of RAI2 expression were detected by semi-quantitative RT-PCR in eight colorectal cancer cell lines. RAI2 was highly expressed in SW480, SW620, DLD1, and DKO cells. Loss of RAI2 expression was detected in RKO, LOVO, and HCT116 cells, and reduced expression of RAI2 was found in HT29 cells (Fig. 1b). The expression of RAI2 was validated by Western blot in protein level in these cells (Fig. 1b).

RAI2 gene promoter region methylation was examined by MSP in these cell lines. Complete methylation was found in RKO, LOVO, and HCT116 cells; unmethylation was found in SW480, SW620, DLD1, and DKO cells, and partial methylation was found in HT29 cells (Fig. 1c). The promoter region methylation is correlated with loss of/reduced expression of RAI2 in colorectal cancer cells. To further validate whether the expression of RAI2 was regulated by promoter region methylation, colorectal cancer cells were treated with DAC, a demethylation agent. Restoration of RAI2 expression was induced by DAC in RKO, LOVO, and HCT116 cells, and increased expression of RAI2 was found in HT29 cells after DAC treatment. No expression changes were found in SW480, SW620, DLD1, and DKO cells after DAC treatment (Fig. 1d). These results suggest that the expression of RAI2 is regulated by promoter region methylation. To further validate the efficiency of MSP primers and the methylation density in the RAI2 promoter region, BSSQ technique was employed (Fig. 1e). Consistent with MSP results, complete methylation was found in LOVO cells, partial methylation was seen in HT29 cells, and SW620 cells were completely unmethylated.

### RAI2 is frequently methylated in primary colorectal cancer, and methylation of RAI2 is associated with poor prognosis

**Fig. 2:**
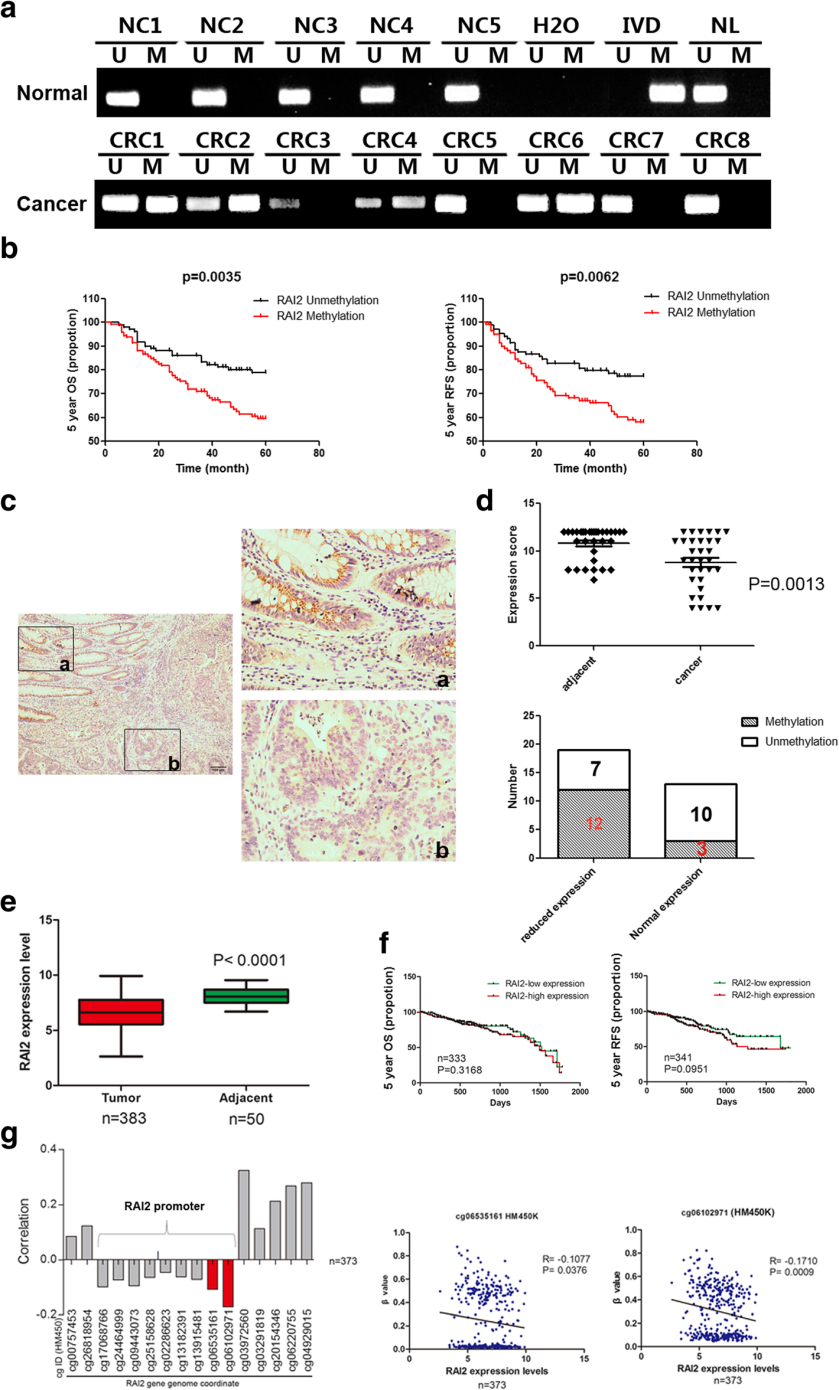
Epigenetic inactivation of RAI2 in primary colorectal cancer. **a** Representative MSP results of RAI2 methylation status in normal colon mucosa (NC) and colorectal cancer tissues (CRC). **b** Kaplan-Meier curves show the association of 5-year overall survival (OS) rate and relapse-free survival (RFS) rate of colorectal cancer patients with the methylation status of RAI2. Black, RAI2-unmethylated colorectal cancer patients (*n* = 110, log-rank test); red, RAI2-methylated colorectal cancer patients (*n* = 127, log-rank test). **c** Representative images of RAI2 protein expression in colorectal cancer and adjacent non-tumor tissues determined by IHC (left images, × 100; right images, × 400). **d** RAI2 expression scores are shown as scatter plots; vertical bars represent the range of data. The expression levels of RAI2 were significantly different between adjacent tissue and colorectal cancer samples (**P < 0.01). The bar diagram shows the expression and DNA methylation status of RAI2 in different cancer samples. Reduced expression of RAI2 was significantly associated with promoter region methylation (**P < 0.01). **e** TCGA data and GTEx data show RAI2 mRNA expression levels in CRC tissues (*n* = 383) and normal colorectal mucosa (*n* = 50) according to RNA-Seq results. Box plots: the levels of RAI2 expression. Horizontal lines: counts of log2 (TPM + 1); TPM, transcripts per million (reads) (***P < 0.001). **f** RAI2 expression and 5-year OS (*n* = 333, log-rank test) and RFS (*n* = 341, log-rank test) from TCGA database for CRC. Kaplan-Meier curves show the association of 5-year OS and 5-year RFS of colorectal cancer patients with RAI2 mRNA expression. Green, RAI2 low-level expression colorectal cancer patients; red, RAI2 high-level expression colorectal cancer patients. **g** The correlation of methylation of 16 CpG sites around the promoter region and expression of RAI2 (upper panel), and the methylation status of the top three CpG sites (cg06102971, cg06535161) are correlated with loss/reduction of RAI2 expression in 373 cases of CRC (all *P* < 0.05).

To explore the methylation status of RAI2 in colorectal cancer, 15 cases of normal colorectal mucosa and 237 cases of primary colorectal cancer samples were analyzed by MSP (Fig. 2a). Methylation was found in 53.6% (127/237) of the colorectal cancer samples, while all 15 cases of normal colorectal mucosa were unmethylated (Fig. 2a). As shown in Table 1, methylation of RAI2 was significantly associated with gender (*P* < 0.001), TNM stage (*P* < 0.001), and lymph node metastasis (*P* < 0.001). No association was found between RAI2 methylation and age, differentiation, tumor location, and size (all *P* > 0.05). Kaplan-Meier analysis indicated that methylation of RAI2 was significantly associated with poor 5-year overall survival (OS) (*P* = 0.0035, Fig. 2b) and 5-year relapse-free survival (RFS) (*P* = 0.0062, Fig. 2b). According to univariate analysis, RAI2 methylation, tumor differentiation, lymph node metastasis, and TNM stage were associated with both poor 5-year OS (all *P* < 0.05) and 5-year RFS (all *P* < 0.05, Table 2). By multivariate analysis, RAI2 methylation, tumor differentiation, and TNM stage were associated with both poor 5-year OS and 5-year RFS (all *P* < 0.05, Table 2). Age, gender, and lymph node metastasis were only associated with poor 5-year OS (all *P* < 0.05, Table 2). These results suggest that RAI2 methylation was an independent prognostic marker for poor 5-year OS (*P* = 0.002, Table 2) and 5-year RFS (*P* = 0.022, Table 2).

**Table 1:** Clinical characteristics and RAI2 methylation status of 237 patients with colorectal cancer

Clinical parameter	RAI2 methylation status		*P* value
	Unmethylated (*n* = 110)	Methylated (*n* = 127)	
Gender			*P* = 0.0000***
Male	95	46	
Female	15	81	
Age (years)			*P* = 0. 5217
≥ 50	87	96	
< 50	23	31	
Tumor location			*P* = 0.2859
Right-sided colon	29	28	
Left-sided colon	36	34	
Rectum	45	65	
Tumor size (cm)			
< 5	61	76	*P* = 0.4951
≥ 5	49	51	
TNM stage			*P* = 0.0001***
I–II	48	26	
III–IV	62	101	
Differentiation			*P* = 0.0841
Middle-high	84	84	
Low	26	43	
Lymph node metastasis			*P* = 0.0000***
Yes	59	101	
No	51	26	
Intravascular cancerous embolus			*P* = 0.0901
Yes	10	21	
No	100	106	

*P* values are obtained from the chi-squared test

Statistically significant *P < 0.05, **P < 0.01, ***P < 0.001

**Table 2:** Analysis of RAI2 methylation status with OS or RFS in colorectal cancer patients by Cox regression analysis

Variables	OS				RFS			
	Univariate analysis		Multivariate analysis		Univariate analysis		Multivariate analysis	
	HR (95% CI)	*P*	HR (95% CI)	*P*	HR (95% CI)	*P*	HR (95% CI)	*P*
RAI2 methylation	0.481	0.004**	0.405	0.002*	0.504	0.008**	0.512	0.022*
M vs U	(0.290–0.796)		(0.226–0.726)		(0.305–0.833)		(0.288–0.907)	
Age (years)	1.913	0.058	0.460	0.027*	1.187	0.567	0.790	0.440
≥ 50 vs < 50	(0.979–3.737)		(0.231–0.915)		(0.659–2.137)		(0.434–1.437)	
Gender	1.013	0.956	1.969	0.018*	1.029	0.907	1.576	0.101
Female vs male	(0.630–1.631)		(1.121–3.455)		(0.635–1.668)		(0.916–2.714)	
Tumor location	0.864	0.592	1.661	0.106	0.986	0.96	1.564	0.176
Distal colon or rectum vs proximal colon	(0.505–1.476)		(0.898–3.070)		(0.563–1.727)		(0.818–2.989)	
Tumor size	0.854	0.519	1.213	0.480	0.759	0.276	1.352	0.299
≥ 5 vs < 5 cm	(0.527–1.381)		(0.710–2.072)		(0.461–1.248)		(0.765–2.388)	
Differentiation	0.396	0.000***	0.460	0.002**	0.369	0.000***	0.449	0.001**
Low vs high/ middle	(0.247–0.634)		(0.283–0.748)		(0.229–0.597)		(0.274–0.735)	
TNM stage	0.262	0.000***	0.089	0.000***	0.237	0.000***	0.069	0.006**
III/IV vs I/II	(0.125–0.546)		(0.027–0.294)		(0.113–0.496)		(0.010–0.461)	
Pathologic N stage	0.418	0.006**	3.985	0.008**	0.283	0.000***	4.505	0.105
N1–2 vs N0	(0.224–0.778)		(1.439–11.034)		(0.140–0.570)		(0.732–27.731)	
Intravascular cancerous embolus	0.597	0.093	0.672	0.220	0.996	0.992	1.095	0.812
Yes vs no	(0.327–1.090)		(0.357–1.267)		(0.476–2.084)		(0.517–2.320)	

*P < 0.05, **P < 0.01, ***P < 0.001

To explore the regulation of RAI2 expression in primary colorectal cancer, RAI2 expression was evaluated by immunohistochemistry (IHC) in 32 cases of matched colorectal cancer and adjacent tissue samples. The expression of RAI2 was reduced significantly in cancer tissue compared to the adjacent normal tissue (*P* < 0.01, Fig. 2c, d). Among the 19 cases of cancer samples that had loss of/reduced expression of RAI2, 12 cases were methylated (63.15%). In contrast, in 13 cases of cancer tissue samples that expressed RAI2, only 3 cases were methylated (23.1%). Loss/reduction of RAI2 expression was significantly associated with promoter region methylation in CRC (*P* < 0.05, Fig. 2d, bottom panel). These results suggest that the expression of RAI2 is regulated by promoter region methylation in human CRC.

To further validate our results, RAI2 mRNA expression and promoter region methylation data were extracted from Genotype-Tissue Expression (GTEx) and The Cancer Genome Atlas (TCGA) (http://xena.ucsc.edu/) databases. RAI2 expression data were obtained from RNA sequencing (RNA-Seq) in 383 cases of CRC samples and 50 cases of adjacent colorectal tissue samples. The levels of RAI2 expression were significantly lower in CRC samples compared to adjacent normal colorectal mucosa samples (*P* < 0.0001, Fig. 2e), while no association was found between RAI2 mRNA expression and 5-year OS (*n* = 333, *P* = 0.3168, Fig. 2f) or 5-year RFS (*n* = 341, *P* = 0.0951, Fig. 2f) in this cohort. Methylation of RAI2 was analyzed by Illumina Infinium Human Methylation 450 (HM450) based on the methylation status of 16 CpGs in the promoter region. Available data were obtained from 373 cases of colorectal cancer samples for both RAI2 expression and methylation. The expression of RAI2 was inversely associated with promoter region methylation (*P* < 0.05; Fig. 2g). These data further support our results. Thus, methylation of RAI2 may serve as a detection and poor prognostic marker in CRC.

### Restoration of RAI2 expression suppresses cell proliferation and induces cell apoptosis in CRC

**Fig. 3:**
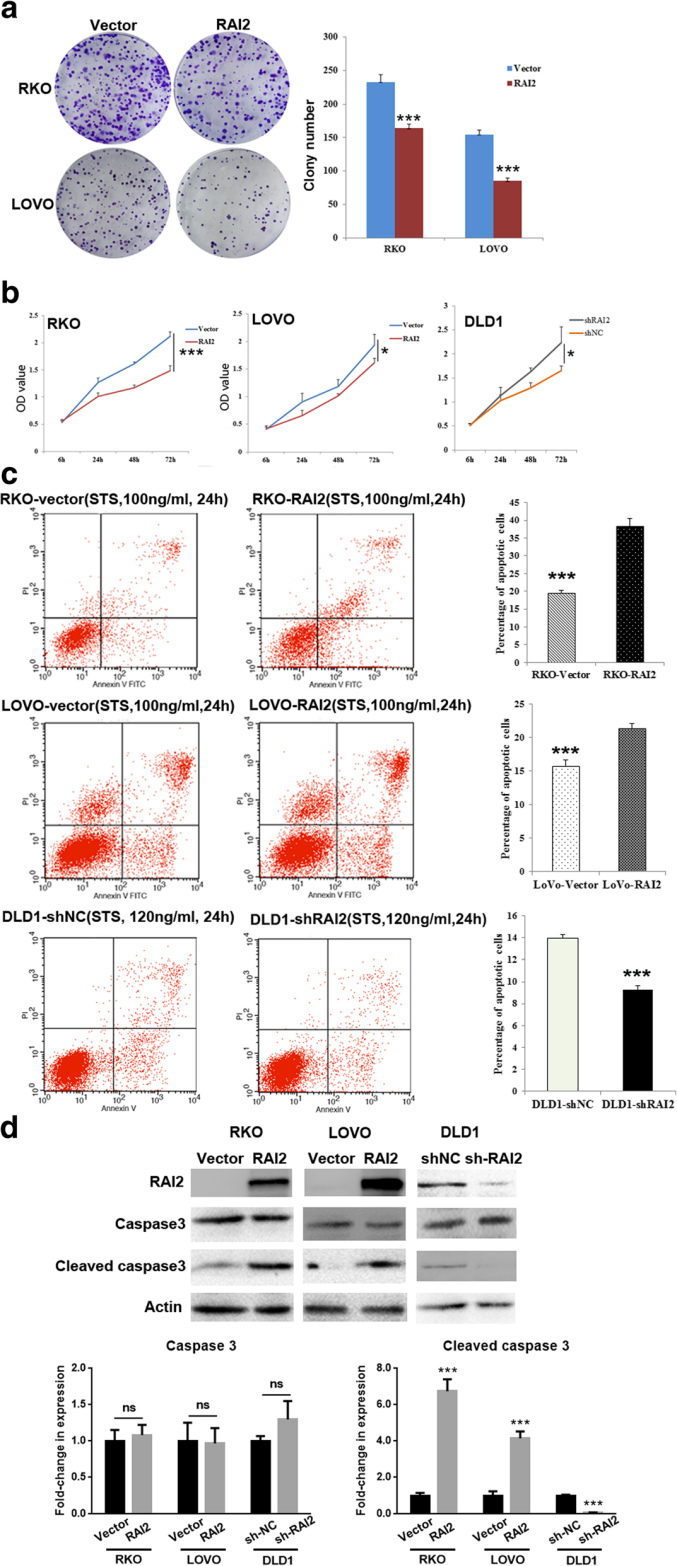
The effect of RAI2 on cell proliferation and apoptosis in colorectal cancer cells. **a** The effects of RAI2 on colony formation in RKO and LOVO cell lines before and after the restoration of RAI2 expression. The experiment was repeated three times (***P < 0.001). **b** Growth curves demonstrate the effect of RAI2 on cell proliferation as measured by the MTT assay for 72 h in RKO and LOVO cell lines before and after the restoration of RAI2 expression, and in DLD1 cell line, before and after knockdown of RAI2. The experiment was repeated three times (***P < 0.001, *P < 0.05). **c** Upper and middle: percentage of apoptotic cells before and after the restoration of RAI2 expression in RKO and LOVO cells under the treatment of STS (100 ng/ml for 24 h). Down: percentage of apoptotic cells and before and after knockdown of RAI2 in DLD1 cells under the treatment of STS (120 ng/ml for 24 h). The experiment was repeated three times (***P < 0.001). **d** Expression levels of RAI2, caspase-3, and cleaved caspase-3 were detected by Western blot before and after the restoration of RAI2 expression in RKO and LOVO cells and before and after knockdown of RAI2 in DLD1 cells. Actin was used as a control.

Colony formation assays were performed to evaluate the effect of RAI2 on clonogenicity. The colony numbers were 233 ± 11 versus 164 ± 6 in RKO cells (*P* < 0.001) and 155 ± 6 versus 85 ± 5 in LOVO (*P* < 0.001) cells before and after the restoration of RAI2 expression, indicating a significant reduction in colony formation upon RAI2 re-expression (Fig. 3a). To further evaluate the effects of RAI2 on cell proliferation, cell viability was detected by MTT assays. The OD values were 2.13 ± 0.08 versus 1.49 ± 0.10 in RKO cells (*P* < 0.001) and 1.93 ± 0.130 versus 1.61 ± 0.08 in LOVO cells (*P* < 0.05) before and after re-expression of RAI2. The OD values were 1.650 ± 0.102 versus 2.239 ± 0.328 (*P* < 0.05) before and after knockdown of RAI2 in DLD1 cells. Cell viability decreased upon restoration of RAI2 expression in RKO and LOVO cells and increased after knockdown of RAI2 in DLD1 cells (Fig. 3b). These results suggest that RAI2 inhibits CRC cell proliferation.

The effects of RAI2 on cell cycle and apoptosis were analyzed by flow cytometry in human colorectal cancer cells. No significant differences in cell cycle phase distribution were found in RKO and LOVO cells before and after re-expression of RAI2 (all *P* > 0.05, data not shown). To increase the sensitivity of apoptosis detection, cultured cells were treated by STS. The ratios of apoptotic cells were 11.16 ± 1.25% versus 20.15 ± 2.75% in RKO cells (*P* < 0.001) and 28.92 ± 2.78% versus 38.12 ± 0.87% in LOVO cells (*P* < 0.001) before and after re-expression of RAI2 under the treatment of STS. The ratio of apoptotic cells increased significantly after re-expression of RAI2 (*P* < 0.001, Fig. 3c). In RAI2 highly expressed DLD1 cells, the ratio of apoptotic cells was 33.76 ± 0.74% before knockdown of RAI2 and 24.83 ± 1.60% after knockdown of RAI2 under STS treatment. The ratio of apoptotic cells decreased significantly (*P* < 0.001, Fig. 3c) after knockdown of RAI2 in DLD1 cells. As shown in Fig. 3d, the levels of cleaved caspase-3 increased after re-expression of RAI2 in RKO and LOVO cells and decreased after knockdown of RAI2 expression in DLD1 cells. The ratio of apoptotic cells was increased by RAI2 under STS treatment in CRC cells. The above results indicate that RAI2 induces apoptosis in CRC cells.

### RAI2 suppresses cell migration and invasion by inhibiting AKT signaling in CRC

**Fig. 4:**
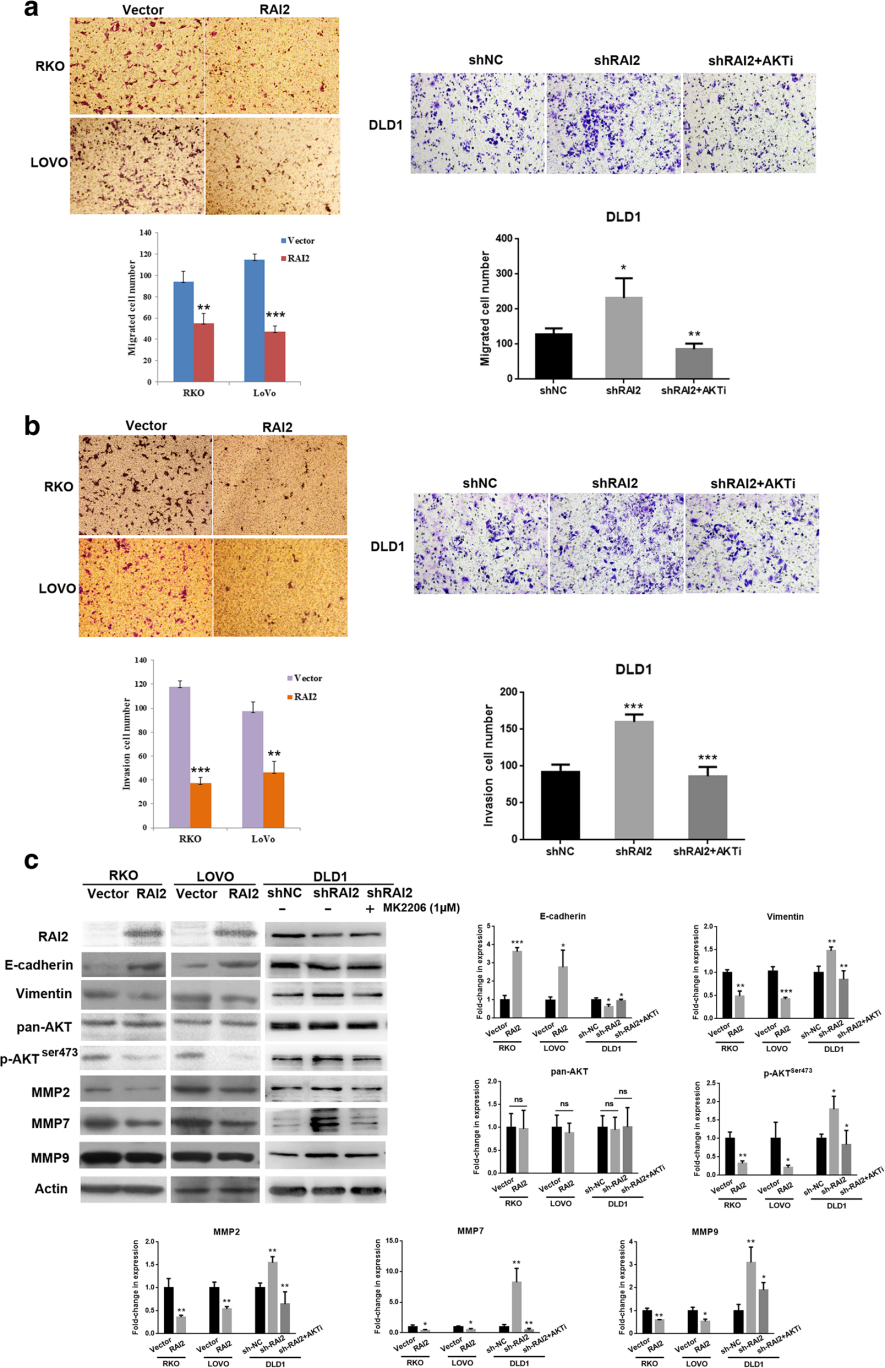
The effect of RAI2 on colorectal cancer migration and invasion. **a** Migration assay results showing the number of migrated cells before and after the restoration of RAI2 expression in RKO and LOVO cells as well as in the control group (shNC), shRAI2 knockdown group, and shRAI2 knockdown plus MK2206 treated group (*P < 0.05, **P < 0.01). **b** Invasion assay results showing the number of invasive cells before and after re-expression of RAI2 in RKO and LOVO cells as well as in the control group (shNC), shRAI2 knockdown group, and shRAI2 knockdown plus MK2206 treated group (***P < 0.001). **c** The expression levels of E-cadherin, vimentin, pan-AKT, p-AKTser473, MMP-2, MMP-7, MMP-9, and actin (control) were detected by Western blot in RAI2 un-expressed and re-expressed RKO and LOVO cells as well as in the control group, shRAI2 knockdown group, and shRAI2 knockdown plus MK2206 treated group (ns, no significance, *P < 0.05, **P < 0.01, ***P < 0.001).

To investigate the role of RAI2 in cell migration and invasion, a transwell assay was employed in RKO and LOVO cells. In RKO cells, the numbers of migrated cells were 94 ± 9.64 and 55 ± 9.17 in the empty vector group and RAI2 re-expressed group for each microscopic field, respectively (*P* < 0.001, Fig. 4a). In LOVO cells, the numbers of migrated cells were 114.67 ± 5.51 and 47.33 ± 5.13 in the empty vector group and RAI2 re-expressed group for each microscopic field, respectively (*P* < 0.001, Fig. 4a). In RAI2 highly expressed DLD1 cells, the numbers of migrated cells for each microscopic field were 127.0 ± 18.08, 231.7 ± 56.50, and 85.67 ± 15.95 in the control group (shNC), shRAI2 knockdown group, and shRAI2 knockdown plus MK2206 treated group, respectively. The numbers of migrated cells were increased significantly in the shRAI2 knockdown group compared to the control group (*P* < 0.05, Fig. 4a). The numbers of migrated cells were reduced significantly after treatment with MK2206 in the shRAI2 knockdown group (*P* < 0.01, Fig. 4a).

Next, the transwell assay with Matrigel coating was employed to evaluate the effect of RAI2 on cell invasion. The numbers of invasive cells for each microscopic field were 187.3 ± 6.5 versus 59.7 ± 5.2 in RKO cells (*P* < 0.01, Fig. 4b) and 117.0 ± 11.2 versus 61.7 ± 5.3 in LOVO cells (*P* < 0.01, Fig. 4b) before and after the restoration of RAI2 expression. In RAI2 highly expressed DLD1 cells, the numbers of invasive cells for each microscopic field were 91.67 ± 10.41, 160.0 ± 10.39, and 86.33 ± 12.58 in the control group, shRAI2 knockdown group, and shRAI2 knockdown plus MK2206 treated group, respectively. The numbers of invasive cells were increased significantly in the shRAI2 knockdown group compared to the control group (*P* < 0.001, Fig. 4b). The numbers of migrated cells were reduced significantly after treatment with MK2206 in the shRAI2 knockdown group (*P* < 0.001, Fig. 4b). These results suggest that cell migration and invasion were suppressed by RAI2 in CRC cells.

Epithelial-mesenchymal transition (EMT) is related to cancer invasion and metastasis [21, 22]. To explore the possible role of RAI2 in EMT, EMT-related markers, E-cadherin, and vimentin were examined by Western blot in RAI2 unexpressed and re-expressed RKO and LOVO cells. The expression of E-cadherin was upregulated, and the expression of vimentin was downregulated by RAI2 in RKO and LOVO cells (Fig. 4c). Metalloproteinases (MMPs) promote cancer cell invasion by degrading extracellular matrix (ECM) [23, 24]. Three representative MMP members, MMP-2, MMP-7, and MMP-9, have been reported to be highly expressed in invasive tumors [25–27]. As shown in Fig. 4c, the expression levels of MMP-2, MMP7, and MMP-9 were reduced after re-expression of RAI2 in RKO and LOVO cells. The expression levels of E-cadherin were reduced, and the levels of vimentin and MMPs were increased after knockdown of RAI2 in DLD1 cells (Fig. 4c). The levels of E-cadherin were increased, and the levels of vimentin and MMPs were decreased after treatment with MK2206 in shRAI2-treated DLD1 cells (Fig. 4c). These results suggest that RAI2 suppresses cell migration and invasion by inhibiting EMT in CRC. It has been reported that depletion of RAI2 activated the AKT signaling cascade in breast cancer [17]. In this study, we found that AKT protein phosphorylation at Ser473 decreased after re-expression of RAI2 in RKO and LOVO cells, phosphorylation of AKT increased after knockdown of RAI2 in RAI2 highly expressed DLD1 cells, and AKT inhibition reverted the phenotype induced by RAI2 depletion. Our results suggest that RAI2 suppresses cell migration and invasion by inhibiting AKT signaling in CRC (Fig. 4c).

### RAI2 suppresses CRC cell xenograft growth in mice

**Fig. 5:**
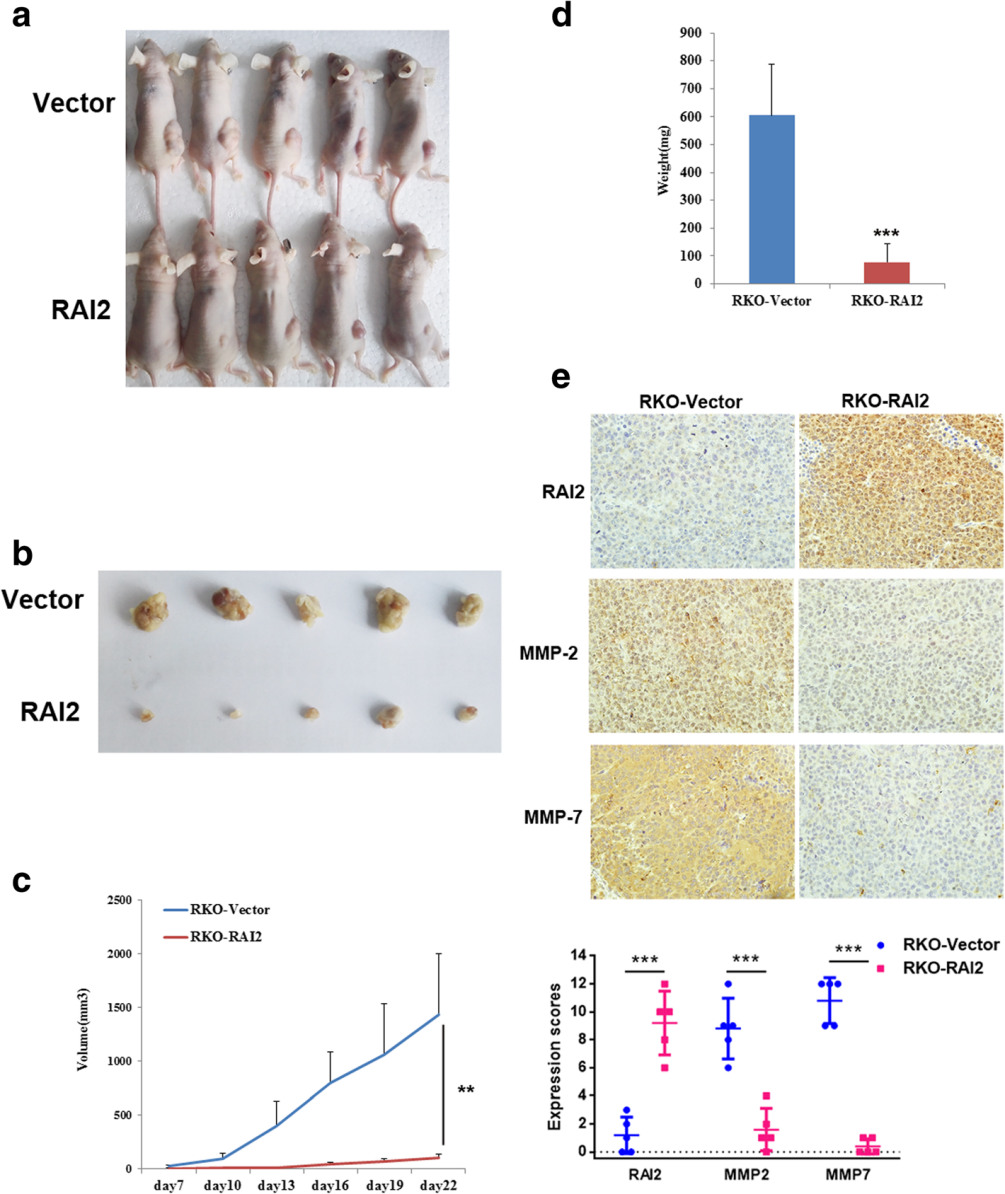
The effect of RAI2 on colorectal cancer cell xenograft mice. **a**, **b** Representative pictures of xenograft tumors from RAI2-unexpressed (upper) and re-expressed RKO cells (lower). **c** Subcutaneous tumor growth curves in xenograft mice with or without RAI2 re-expression (**P < 0.01). **d** Histogram represents the average weight of xenograft tumors in RAI2 unexpressed and re-expressed groups (***P < 0.001). **e** IHC staining reveals the expression levels of RAI2, MMP2, and MMP7 in RAI2-unexpressed and RAI2 re-expressed-RKO cell xenografts (× 400). The expression scores are shown below as scatterplots (***P < 0.001).

To further explore the effects of RAI2 in CRC, a human colorectal cancer cell xenograft mouse model was employed. As shown in Fig. 5a–c, the average tumor volume was 105.09 ± 34.57 mm^3^ in RAI2 expressed RKO cell xenografts, and the average tumor volume was 1428.26 ± 566.46 mm^3^ in RAI2 unexpressed RKO cell xenografts. The tumor volume was significantly reduced after the restoration of RAI2 expression in RKO cells (*P* < 0.01). The tumor weight was 78.60 ± 66.83 mg in RAI2-expressed RKO cell xenografts and 606.00 ± 182.70 mg in RAI2-unexpressed RKO cell xenografts. The tumor weight was reduced significantly after the restoration of RAI2 expression in RKO cells (*P* < 0.001, Fig. 5d). The expression of MMP2 and MMP7 was inhibited by RAI2 in RKO cell xenografts Fig. 5e. These results suggest that RAI2 suppresses CRC cell tumor growth in vivo.

## Discussion

There has only been one report addressing RAI2 in human cancers [17]. By using six publicly available datasets, the authors found that lower RAI2 transcript expression was associated with shortened OS in breast, lung, ovarian, and colonic cancer. In our study, according to the analysis using The Human Protein Atlas database, RAI2 was downregulated in human CRC, and based on the TCGA database, the RAI2 gene was rarely mutated in CRC. Then, we screened the expression of RAI2 in CRC cells and primary cancer samples. The loss of RAI2 expression was found frequently in CRC cells, and the expression of RAI2 was reduced significantly in cancer tissue compared to the adjacent normal tissue samples. These results suggest that RAI2 may be a tumor suppressor and diagnostic marker in CRC. RAI2 is rarely mutated in human CRC according to the TCGA database analyzing, while the expression of RAI2 was frequently lost/reduced in CRC cell lines and tissue samples. Thus, we explored the epigenetic regulation and function of RAI2 in human CRC. As our expectation, RAI2 was validated to be regulated by promoter region methylation in CRC. RAI2 was frequently methylated in human primary CRC, and methylation of RAI2 was significantly associated with female gender, TNM stage, and lymph node metastasis. There is no report about the association of RAI2 expression or methylation with gender. Werner et al. found that RAI2 expression was highest in the epithelial-like ER^+^ cell lines, whereas its expression was lost in the mesenchymal-like and highly metastatic cell lines [17]. Interestingly, treatment with either ER antagonists or retinoic acid could induce RAI2 expression [17]. Therefore, it is important to further understand how retinoic acid and RAI2 influence estrogen signaling in the future [28]. RAI2 methylation may be related to CRC progression and metastasis. Further analysis suggested that methylation of RAI2 is significantly associated with poor 5-year overall survival and 5-year relapse-free survival (RFS). According to Cox proportional hazards model analysis, RAI2 methylation was an independent prognostic marker for poor 5-year OS and poor 5-year RFS. This is the first report on the epigenetic regulation of RAI2 and its clinical relevance in CRC. As DNA is very stable, it is much easier and more reproducible to detect DNA methylation than to detect the expression of mRNA and protein in human primary cancer. Thus, RAI2 methylation may serve as a prognostic marker in human CRC.

To further understand the role of RAI2 in CRC development, we analyzed the function of RAI2 both in vitro and in vivo. RAI2 suppressed CRC cell proliferation, migration, and invasion, as well as induced cell apoptosis. RAI2 inhibited the AKT signaling pathway in CRC cells and suppressed CRC cell xenograft growth. These results suggest that RAI2 is a tumor suppressor in human CRC, and methylation of RAI2 is a potential epigenetic therapeutic target.

## Conclusion

RAI2 is frequently methylated in human CRC, and the expression of RAI2 is regulated by promoter region methylation. Methylation of RAI2 is significantly associated with poor 5-year OS and 5-year RFS, and it is an independent prognostic marker of poor 5-year OS and poor 5-year RFS. RAI2 suppresses CRC cell growth both in vitro and in vivo. RAI2 induces cell apoptosis and suppresses CRC cell migration and invasion. RAI2 may serve as a tumor suppressor by inhibiting the AKT signaling pathway in CRC.

## Abbreviations

BSSQ: Bisulfite sequence
CRBP1: Retinol-binding protein 1
CRC: Colorectal cancer
DAC: 5-Aza-2′-deoxycytidine
ECM: Extracellular matrix
EMT: Epithelial-mesenchymal transition
GAPDH: Glyceraldehyde-3-phosphate dehydrogenase
GTEx: Genotype-Tissue Expression
HM450: Illumina Infinium Human Methylation 450
IHC: Immunohistochemistry
IVD: In vitro-methylated DNA
MMPs: Metalloproteinases
MSP: Methylation-specific PCR
NHS: Nance-Horan syndrome
NL: Normal lymphocyte DNA
OS: Overall survival
RA: Retinoic acid
RAI2: Retinoic acid-induced 2
RARβ2: Retinoic acid receptor β2
RFS: Relapse-free survival
RNA-Seq: RNA sequencing
STS: Staurosporine
TCGA: The Cancer Genome Atlas
TSS: Transcription start site
